# Effects of *Wx* Genotype, Nitrogen Fertilization, and Temperature on Rice Grain Quality

**DOI:** 10.3389/fpls.2022.901541

**Published:** 2022-07-22

**Authors:** Duo Xia, Yipei Wang, Qingyun Shi, Bian Wu, Xiaoman Yu, Changquan Zhang, Yanhua Li, Pei Fu, Minqi Li, Qinglu Zhang, Qiaoquan Liu, Guanjun Gao, Hao Zhou, Yuqing He

**Affiliations:** ^1^National Key Laboratory of Crop Genetic Improvement, Hubei Hongshan Laboratory, Huazhong Agricultural University, Wuhan, China; ^2^Key Laboratory of Plant Functional Genomics of the Ministry of Education, Jiangsu Key Laboratory of Crop Genomics and Molecular Breeding, College of Agriculture, Yangzhou University, Yangzhou, China; ^3^State Key Laboratory of Crop Gene Exploration and Utilization in Southwest China, Rice Research Institute, Sichuan Agricultural University, Chengdu, China

**Keywords:** amylose content, appearance quality, eating quality, protein content, *Wx* genotype, nitrogen fertilization, temperature

## Abstract

Quality is a complex trait that is not only the key determinant of the market value of the rice grain, but is also a major constraint in rice breeding. It is influenced by both genetic and environmental factors. However, the combined effects of genotypes and environmental factors on rice grain quality remain unclear. In this study, we used a three-factor experimental design to examine the grain quality of different *Wx* genotypes grown under different nitrogen fertilization and temperature conditions during grain development. We found that the three factors contributed differently to taste, appearance, and nutritional quality. Increased *Wx* function and nitrogen fertilization significantly reduced eating quality, whereas high temperature (HT) had almost no effect. The main effects of temperature on appearance quality and moderate *Wx* function at low temperatures (LTs) contributed to better appearance, and higher nitrogen fertilization promoted appearance at HTs. With regard to nutritional quality, *Wx* alleles promoted amylose content (AC) as well as starch-lipids content (SLC); nitrogen fertilization increased storage protein content (PC); and higher temperature increased lipid content but decreased the PC. This study helps to broaden the understanding of the major factors that affect the quality of rice and provides constructive messages for rice quality improvement and the cultivation of high-quality rice varieties.

## Introduction

As one of the major food crops worldwide, rice is consumed as a staple food by almost half of the world population and provides about 20% of the total caloric intake (Fan et al., 2012). With improved living standards, the demand for higher rice cooking and eating quality has increased (Tan et al., 2002). Consumers show a preference for varieties that have superior quality, and improving quality is a current focus on breeding programs second only to yield enhancement (Zhang, 2007).

Rice quality encompasses several characteristics, such as milling, appearance, cooking, and eating (Fitzgerald et al., 2009). It is a complex trait affected by both genetic and environmental factors. Numerous studies on the genetic and molecular bases of rice quality have led to significant achievements. Grain shape, a main component of appearance, is controlled by major genes, such as *GS3* and *GW5*, and multiple minor genes, such as *GL3.1*, *GL3.3*, and *GW7* (Wang et al., 2015; Li et al., 2018; Sun et al., 2018; Xia et al., 2018). *Chalk5* and *WCR1* were reported to affect the grain chalkiness, an undesirable attribute of appearance quality (Li et al., 2014; Wu et al., 2022). *Wx*, the major gene that controls amylose content (AC), is responsible for the wide diversity of cooking and eating qualities, with the individual alleles affecting gel consistency (GC), viscosity, and taste (Wang et al., 2007; Zhou et al., 2021b). *ALK* and *fgr* are major genes that determine gelatinization temperature and aroma, respectively (Chen et al., 2008; Gao et al., 2011). Natural variation in *OsGluA2* and *OsAAP6* is associated with protein content (PC), a determinant of nutritional quality (Peng et al., 2014; Yang et al., 2019).

Environmental factors, such as light, carbon dioxide concentration, temperature, nutrients, and water, also affect rice quality (Philpot et al., 2006; Patindol et al., 2015). Among these factors, the effects of temperature and nitrogen fertilization were intensively studied because of the threat of global warming on food security and for the maintenance of yield at higher temperatures (Peng et al., 2004). High temperatures (HTs) were associated with deterioration in milling quality and lower cooking and eating qualities (Counce et al., 2005; Zhong et al., 2005). Lin et al. (2010) found that HTs (35/30°C day/night) reduced grain weight, AC, and flour GC but led slightly increased glutelin and albumin contents. Zhang et al. (2016) pointed out that HTs during grain filling induced chalkiness and reduced AC, leading to poor milling quality and reduced yield. The changes in AC and starch structure were the main reason for the effect of HT on rice quality (Fan et al., 2019; Zhang H. et al., 2021). Nitrogen fertilization affects the rice quality through its influence on PC and starch properties (Yang et al., 2016; Zhu et al., 2016, 2017). Singh et al. (2011) reported that nitrogen fertilizer application decreased AC in rice grain and Gu et al. (2015) found that eating quality declined with the application of nitrogen fertilizer. Cao et al. (2018) pointed out that the application of fertilizer at later growth stages could cause increased AC and reduced amylopectin branching and lead to changed starch pasting properties and reduced cooking quality.

From the perspective of improving the rice quality, both genetic and environmental factors must be considered. However, the effects on the quality of combined genetic and environmental factors have not been adequately investigated. The present study was undertaken to investigate the effects of *Wx* genotype, nitrogen fertilization, and temperature on rice quality. The results contribute to knowledge regarding key factors that affect quality under field conditions and provide theoretical information for improving quality in practice.

## Materials and Methods

### Plant Materials and Experimental Design

A series of *Wx* near-isogenic lines (NILs) and *Wx* transgenic lines carrying different *Wx* alleles were used as the test materials (Supplementary Table 3). Details of their construction were described previously (Zhang et al., 2019; Zhang C. Q. et al., 2021; Zhou et al., 2021b). The materials were planted at Huazhong Agricultural University Farm (N30.49°, E114.36°) in the 2020 rice cropping season.

**FIGURE 1:**
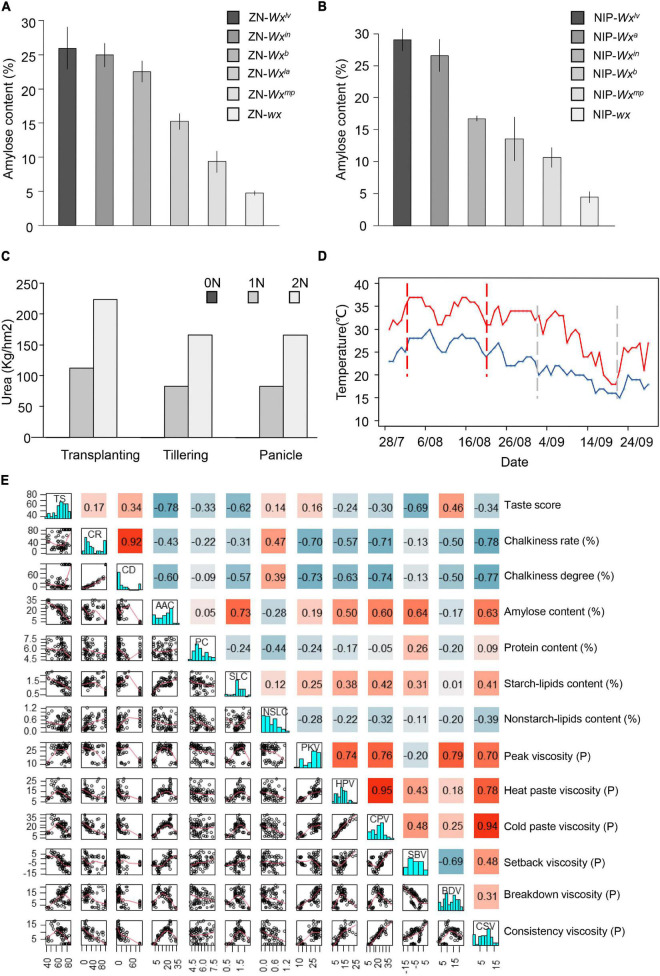
Phenotypic analysis. **(A,B)** Grain amylose content (AC) transformants and near-isogenic lines (NILs). **(C)** The urea application density in field. **(D)** Profiles of the highest (red line) and the lowest (blue line) daily temperatures during grain filling of the rice lines. The space between the vertical dashed red lines represents the flowering period under high temperature (HT) conditions and the area between the vertical dashed gray lines represent the flowering period under low temperature conditions. **(E)** Correlation matrix among quality traits. P is short for Pascal, the unit of pressure.

About 150 seeds were sown on 20 May and 20 June, and seedlings were transplanted to the field on 10 June and 13 July, respectively, to examine the effects of HTs on quality. The materials began to flower on 1 August and 31 August. The NILs were about 2 days earlier than the *Wx* transgenics reflecting the difference between the recipient genotypes. Daily temperature and weather conditions during the flowering to harvest period were recorded (Figure 1D and Supplementary Table 2). Nitrogenous fertilization was arranged in a split plot design with the fertilizer level as the main plot and variety as the split plot. Urea was applied at 0, 125, and 250 kg/ha (0, 1, and 2 N, respectively) in three applications; 40% at transplanting, 30% at tillering, and 30% at heading (Figure 1C). Each of the 6 plots was 42 m^2^ (14 m × 3 m) and the planting density was 16.5 cm between plants with a row spacing of 26 cm. Agronomic management followed local recommendations.

Each line was planted in three replications with 36 plants (three rows of 12 plants). Plants in the inner row were harvested to avoid edge effects. Harvested grains were air-dried and stored at room temperature before trait measurement. For each line grown under different conditions, at least four samples were randomly chosen for traits measurement and each sample was repeated two times.

### Measurements of Eating and Appearance Qualities

Mature seeds for eating quality tests were dried in an oven (37°C) for 24 h prior to husking and milling. For the measurement of AC and pasting properties, polished grains were ground into flour and passed through 200-mesh sieve; 0.01 g of flour was used to measure AC using the I_2_-KI method (Bao et al., 2006). In addition, 3 g of flour was weighed and assessed on a rapid visco-analyzer (Perten, RVA4500) to obtain the pasting properties according to the method of Bao et al. (2006). Furthermore, 30 g of polished rice grains were washed, 40.5 ml of water was added, and after soaking for 30 min, the sample was cooked for 40 min on a steamer pot. After cooling, 8 g of cooked rice was made into rice balls according to recommended procedures and assessed by a taste analyzer kit (Satake, STA1B-RHS1A-RFDM1A, Japan) that included a taste analyzer, a freshness meter, and a hardness and viscosity analyzer to get taste scores. About 10 g of polished rice grains were placed separately on a high-resolution scanner (MICROTEK ScanMaker i800 plus) and the chalkiness ratio, chalkiness degree (CD), and transparency were assessed by a Wanshen SC-E rice grain appearance inspection and analysis system according to methods NY/T 593–2013 and GB/T 17891–1999 (Peng et al., 2018).

### Measurements of Nutrition Traits

Four kinds of storage protein, namely, albumin, globulin, prolamin, and glutelin, were extracted and measured as described (Chen et al., 2018); 0.02 g of defatted milled rice flour was placed into a 2 ml centrifuge tube and a total of 2 ml 0.01 M Tris-HCl (PH 7.5) solution, 1 M NaCl solution, 70% ethanol alcohol, and 0.05 M NaOH solution were used in turn to extract albumin, globulin, prolamin, and glutelin, respectively. Extractions of albumin and globulin were repeated three times, extraction of prolamin repeated two times, and glutelin extraction was carried out four times. The concentrations of the final supernatants were determined by the Coomassie brilliant blue G-250 dye-binding method (Bradford, 1976). Bovine serum albumin was used as a standard, and absorbance at 595 nm measured on an Infinite M200 (Tecan Group, Männedorf, Switzerland) spectrophotometer was used to analyze and calculate the storage PC.

The lipids and starch-lipids were extracted and measured using gas chromatography–mass spectrometry (GC-MS) to measure fatty acid methyl esters (FAMEs) as described (Zhou et al., 2021a); 4 g of milled rice was ground into flour and passed through an 80-mesh sieve and dried in an oven (80°C) for 24 h before lipid or starch-lipids extraction. For lipid extraction, 4.5 ml sulfuric acid:methanol (1:20) solution was added to a 10 ml glass tube with 1.0 g milled rice flour, and 100 μl margaric acid (C17:0) solution (43.94 mg C17:0 dissolved in 10 ml chloroform) was added as an internal standard. The mixture was water-bathed for 3 h at 88°C. After cooling to room temperature, 2 ml ddH_2_O and 2 ml *n*-hexane were added in turn before centrifuging to extract FAMEs. The FAMEs were analyzed by GC-MS as described (Zhou et al., 2021a). For starch-lipids extraction, 4 ml chloroform:methanol (2:1) solution was added to remove non-starch lipids. After three cycles of supernatant removal, the residue flour was used to extract starch-lipids following the same procedure.

### Statistical Analysis

Bar plots, histograms, box plots, and correlations were constructed using phenotypic grand means for NIL or transformant. The *p*-values for Pearson’s correlation coefficients were calculated by two-tailed *t*-*tests* using the ggcorr() function from R package “GGally” (Ihaka and Gentleman, 1996; Ginestet, 2011). Univariate and multivariate analyses of variance were performed using aov() function in R. Multiple comparisons of the effects of each *Wx* allele or nitrogen treatment were computed using the LSD.test() function with the Bonferroni correction from R package “agricolae” (Ihaka and Gentleman, 1996).

## Results

### Phenotypic Variation and Correlations Among Quality Traits

The average maximum/minimum temperatures during grain filling of two plantings were 34.5/27.0°C and 26.4/19.0°C (Figure 1D). As shown in Table 1, appearance quality, eating quality, and nutritional quality varied with *Wx* genotype, temperature, and level of nitrogen application. HTs resulted in significantly lower appearance quality (*p* = 1.71E-04), and slightly decreased AC and storage PC (*p* = 1.42E-08). Pasting properties, such as peak viscosity (PKV), cold paste viscosity (CPV), setback viscosity (SBV), breakdown viscosity (BDV), and consistence viscosity (CSV), varied at different temperatures. There was a significant increase (*p* = 8.77E-04) in starch-lipid contents (SLC) and non-starch-lipid contents (NSLC) at HTs. Nitrogen fertilization significantly increased storage PC at the cost of taste value. Nitrogen application led to a slight improvement in appearance quality but had no effect on AC or lipid contents. The *Wx* genotype affected almost every quality trait (Table 1 and Supplementary Table 1). AC, chalkiness rate (CR), and CD decreased with falling *Wx* function, whereas taste score increased.

**TABLE 1:** Phenotypic variation*^o^* of the tested materials at different temperatures and nitrogen management.

	Taste score	CR (%)	CD (%)	AC (%)	PC (%)	SLC (%)	NSLC (%)	PKV (P)	HPV (P)	CPV (P)	SBV (P)	BDV (P)	CSV (P)
**Temperature Treatment*^p^***													
HT	68.7 ± 10.7	54.2 ± 31.7	31.4 ± 33.6	16.8 ± 8.4	5.3 ± 0.6	1.6 ± 0.4	0.7 ± 0.3	25.9 ± 8.1	13.4 ± 5.1	20.9 ± 8.3	-4.9 ± 5.4	12.5 ± 5.9	7.6 ± 3.7
LT	66.0 ± 10.9	36.1 ± 31.6**	22.8 ± 35.4**	18.8 ± 10.2	6.4 ± 0.9**	1.3 ± 0.3**	0.2 ± 0.2**	24.8 ± 7.2	13.4 ± 4.5	22.6 ± 8.8	-2.2 ± 5.6*	11.4 ± 4.4	9.2 ± 4.7
**Nitrogen Treatment*^q^***													
0N	72.0 ± 10.7 a	46.7 ± 34.8	28.1 ± 35.2	18.0 ± 9.4	5.0 ± 0.4 a	1.4 ± 0.4	0.47 ± 0.38	27.2 ± 8.0	14.3 ± 4.9	22.7 ± 8.5	–4.6 ± 6.1	13.0 ± 56.0	8.4 ± 4.2
1N	66.2 ± 10.3 b	46.1 ± 31.5	27.3 ± 34.6	18.0 ± 9.9	5.9 ± 0.7 b	1.4 ± 0.4	0.44 ± 0.26	25.3 ± 7.3	13.3 ± 4.8	21.9 ± 8.9	–3.4 ± 5.8	12.0 ± 4.9	8.6 ± 4.7
2N	63.7 ± 10.1 c	42.8 ± 33.0	25.8 ± 35.2	17.4 ± 9.0	6.6 ± 0.9 c	1.4 ± 0.4	0.44 ± 0.26	23.4 ± 7.4	12.5 ± 4.7	20.8 ± 8.4	–2.7 ± 5.0	10.9 ± 4.6	8.2 ± 4.1
***Wx* Genotype*^r^***													
Nip-*Wx^lv^*	43.7 ± 3.6 h	63.1 ± 18.0 a	27.4 ± 12.5 a	32.0 ± 3.6 a	6.0 ± 1.0	2.1 ± 0.2 a	0.6 ± 0.3	11.9 ± 1.1 b	8.4 ± 1.1 f	13.4 ± 1.2 g	1.4 ± 0.5 b	3.5 ± 0.7 g	4.8 ± 1.1 de
Nip-*Wx^a^*	54.4 ± 5.2 g	34.9 ± 28.6 bc	11.6 ± 11.2 bc	26.8 ± 0.7 b	5.7 ± 1.0	1.6 ± 0.1 bc	0.4 ± 0.2	31.3 ± 1.6 b	23.5 ± 2.3 a	36.7 ± 3.6 a	5.4 ± 2.5 a	7.8 ± 1.7 f	13.2 ± 3.7 a
Nip-*Wx^in^*	61.9 ± 7.1 f	30.2 ± 13.4 bcd	9.7 ± 5.6 bc	22.0 ± 3.9 cd	5.8 ± 1.2	1.6 ± 0.2 b	0.5 ± 0.3	27.2 ± 2.6 a	16.5 ± 2.8 b	30.4 ± 3.2 b	3.2 ± 3.6 ab	10.7 ± 2.6 e	13.9 ± 2.2 a
Nip-*Wx^b^*	71.5 ± 4.4 cd	13.5 ± 10.1 d	3.3 ± 2.8 c	19.5 ± 4.3 de	5.8 ± 0.9	1.6 ± 0.3 b	0.5 ± 0.4	29.6 ± 3.0 a	15.9 ± 1.1 bc	27.5 ± 1.4 c	–2.1 ± 2.2 c	13.7 ± 2.2 cd	11.6 ± 0.8 ab
Nip-*Wx^mp^*	76.6 ± 5.5 abc	30.8 ± 6.4 bcd	7.9 ± 1.9 bc	9.7 ± 0.7 f	5.7 ± 1.0	1.3 ± 0.3 cd	0.5 ± 0.2	30.4 ± 2.6 a	11.5 ± 1.2 e	18.2 ± 1.9 f	–12.2 ± 2.1e	18.9 ± 1.7 a	6.7 ± 1.0 cd
Nip-*wx*	77.8 ± 6.4 ab			4.2 ± 1.2 g	5.9 ± 1.1	1.0 ± 0.2 e	0.7 ± 0.3	12.8 ± 2.8 a	7.0 ± 1.5 f	8.8 ± 1.7 h	–4.0 ± 1.0 d	5.9 ± 1.3 f	1.8 ± 0.2 ef
ZN-*Wx^lv^*	63.5 ± 2.7 ef	64.8 ± 29.2 a	27.7 ± 16.0 a	27.1 ± 1.5 b	5.7 ± 1.2	1.6 ± 0.3 b	0.3 ± 0.2	26.2 ± 4.7 a	14.2 ± 2.2 cd	24.1 ± 1.5 de	–2.1 ± 5.8 c	12.0 ± 5.7 de	9.9 ± 1.2 b
ZN-*Wx^in^*	68.3 ± 5.0 de	18.0 ± 6.6 cd	6.4 ± 2.8 bc	24.4 ± 1.8 bc	6.0 ± 1.1	1.5 ± 0.2 bcd	0.3 ± 0.3	30.2 ± 3.0 a	14.9 ± 1.6 bc	26.5 ± 1.3 cd	–3.7 ± 2.6 cd	15.3 ± 2.5 bc	11.6 ± 0.5 ab
ZN-*Wx^b^*	69.2 ± 5.1 d	16.1 ± 1.6 cd	5.3 ± 1.1 c	19.9 ± 1.0 cd	5.9 ± 1.0	1.5 ± 0.3 bcd	0.3 ± 0.3	31.3 ± 2.7 a	15.2 ± 1.7 bc	26.4 ± 1.7 cd	–4.9 ± 1.6 cd	16.1 ± 1.8 bc	11.2 ± 1.1 ab
ZN-*Wx^la^*	70.0 ± 4.4 d	28.8 ± 11.3 d	9.1 ± 4.7 bc	15.1 ± 1.1 e	5.9 ± 0.9	1.4 ± 0.2 bcd	0.4 ± 0.2	29.0 ± 2.5 a	13.9 ± 1.5 cd	22.5 ± 1.1 e	–6.5 ± 1.7 d	15.1 ± 1.1 bc	8.6 ± 0.7 bc
ZN-*Wx^mp^*	72.6 ± 3.6 bcd	41.9 ± 23.1 b	16.3 ± 13.1 b	9.8 ± 1.5 f	5.9 ± 0.9	1.3 ± 0.1 d	0.4 ± 0.3	29.2 ± 3.3 a	12.3 ± 3.2 de	18.1 ± 4.2 f	–11.1 ± 3.0 e	16.9 ± 2.7 ab	5.8 ± 1.2 cd
ZN-*wx*	78.4 ± 3.6 a			3.0 ± 1.0 g	5.9 ± 0.8	0.6 ± 0.1 e	0.6 ± 0.4	15.0 ± 1.7 b	7.2 ± 1.0 f	8.9 ± 1.2 h	–6.2 ± 0.6 d	7.9 ± 0.8 f	1.7 ± 0.2 f

*^o^Data are expressed as the mean ± standard deviation (SD), n > 12.*

^p^ * and ** indicates p < 0.01 and p < 0.005 basing on two-tailed t-tests.

***^q^** a–c, indicate significant differences at p < 0.01, using Turkey’s multiple-comparison tests.*

***^r^** a, b, c, d, e, f, g, and h were calculated using the Bonferroni multiple-comparison tests.*

Correlation analyses showed that the taste score was most highly correlated with AC (*r^2^* = -0.78) followed by SBV and starch-lipids content (SLC) (Figure 1E). AC was positively correlated with starch-lipid content and most viscosity traits except BDV, and opposite to that between PC and viscosity traits (Figure 1E). The CR and CD were closely correlated with each other and negatively correlated with AC and most viscosity properties (Figure 1E). These results indicated that AC was the key determinant of the taste score. Storage PC, lipids content, and appearance quality also affected the eating quality.

### *Wx* Genotype Affects Eating Quality and Appearance Quality

**FIGURE 2:**
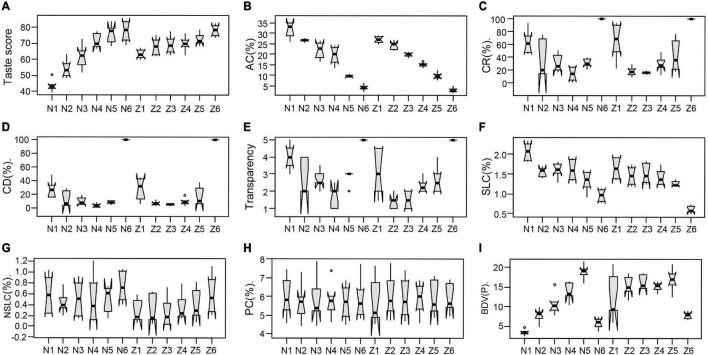
Effects of *Wx* genotype on quality traits. **(A)** Taste score, **(B)** AC, **(C)** chalkiness rate (CR), **(D)** chalkiness degree (CD), **(E)** transparency, **(F)** starch-lipids content (SLC), **(G)** non-starch-lipids content (NSLC), **(H)** grain protein content (PC), and **(I)** breakdown viscosity (BDV). N1–N6 are NILs Nip-*Wx^lv^*, Nip-*Wx^a^*, Nip-*Wx^in^*, Nip-*Wx^b^*, Nip-*Wx^mp^*, and Nip-*wx*; and Z1–Z6 are transformants ZN-*Wx^lv^*, ZN-*Wx^in^*, ZN-*Wx^b^*, ZN-*Wx^la^*, ZN-*Wx^mp^*, and ZN-*wx* lines.

**FIGURE 3:**
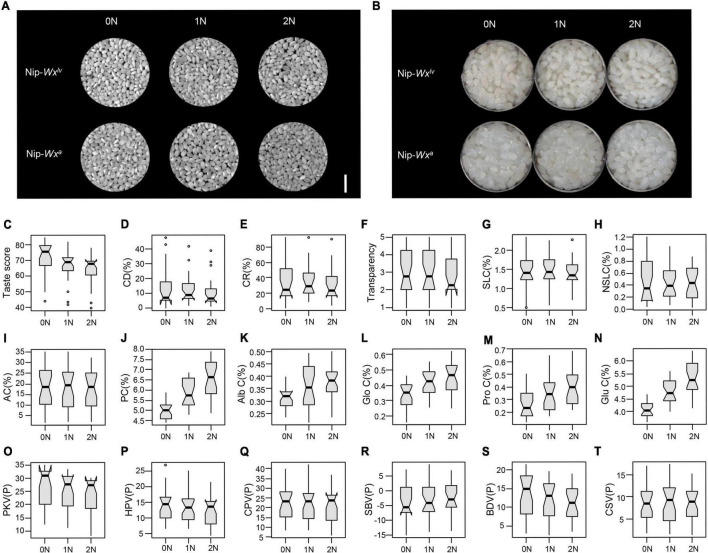
Effects of nitrogen fertilization level on rice quality traits. **(A,B)** Grain appearance **(A)** and cooked rice appearance **(B)** of Nip-*Wx^lv^* and Nip-*Wx^a^* at different nitrogen fertilization levels. **(C–T)** The mean effects of nitrogen fertilization level on **(C)** taste score; **(D)** CD; **(E)** CR; **(F)** transparency; **(G)** SLCs; **(H)** NSLCs; **(I)** AC; **(J)** PC; **(K)** albumin content; **(L)** globulin content; **(M)** prolamin content; **(N)** glutelin content; and **(O–T)** viscosity properties.

*Wx*, which encodes the GBSSI, is the major gene affecting AC in rice (Mikami et al., 2008; Zhang et al., 2019). The ACs of the NILs and transgenic lines varied with *Wx* alleles (Figures 1A,B, 2A and Table 1). Taste score increased with the decreased AC and *Wx* function, consistent with the negative correlation between AC and taste score (Figures 1E, 2A,B). There were larger effects of *Wx* alleles on taste score in Nipponbare than in Zhennuo (Figure 2A). In addition, the *Wx* genotype was associated with grain chalkiness, but the effects were irregular (Figures 2C, 3A and Supplementary Figure 1B). The CR decreased with *Wx* function but the CRs of glutinous *wx* allele or soft rice *Wx* alleles (*Wx^mp^* and *Wx^la^*) were inconsistent in the opaque appearance of these alleles (Figures 2A,C, 3A). A relatively superior appearance quality (with low CR and degree) was observed in lines carrying the *Wx^b^* allele (Figures 2C,D, 3A). The *Wx* genotype was associated with the starch-lipid contents (Figure 2F). The change of starch-lipid contents in transgenic lines and NILs was consistent with changes in AC, evidenced by a positive correlation between starch-lipid contents and *Wx* function and with the relationship between AC and SLC (Figures 1E, 2B,F). Taking these results together, we concluded that the *Wx* locus has multiple effects on quality.

### Effects of N-Level on Nutritional Quality, Eating Quality, and Appearance Quality

Many quality traits varied with the level of nitrogen application (Table 1 and Figures 3C–T). The most obvious change was increased storage PC with increased nitrogen fertilization (Table 1 and Figures 3J–N). However, nitrogen fertilization significantly decreased the taste scores without influencing the AC (Figure 3C). Pasting properties also changed with the nitrogen fertilization level (Figures 3O–T and Table 1). There was a slight improvement in grain appearance and the cooked rice appearance was witnessed in high amylose lines under HT (Figures 3A,B and Supplementary Figures 1C,D). The CR and CD slightly decreased as the nitrogen level increased, and cooked rice from the high nitrogen fertilization level was whiter and less glossy than that from low nitrogen fertilization (Figures 3A,B). Thus, nitrogen fertilization improved PC and slightly improved grain appearance, but at the cost of eating quality.

### Effects of Temperature on Various Aspects of Quality

**FIGURE 4:**
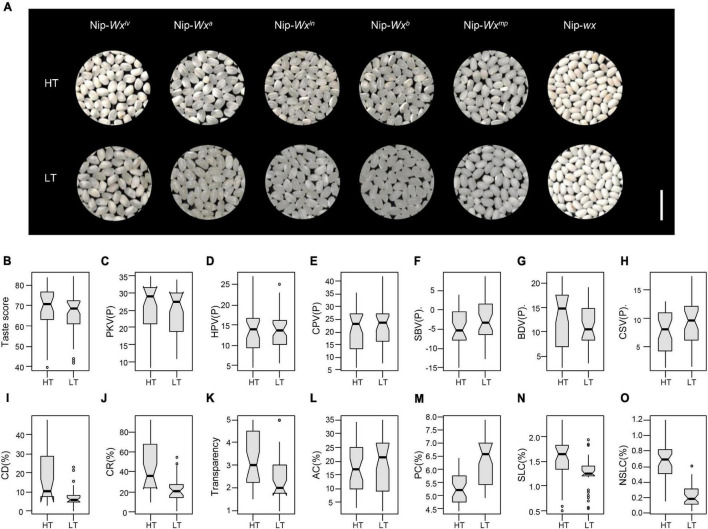
Effects of temperature on appearance quality and other quality traits. **(A)** Grain appearance of Nip-*Wx^lv^*, Nip-*Wx^a^*, Nip-*Wx^in^*, Nip-*Wx^b^*, Nip-*Wx^mp^*, Nip-*wx* at high (HT) and normal (LT) temperatures. The mean effects of temperature on **(B)** taste score; **(C–H)** viscosity properties; **(I)** CD; **(J)** CR; **(K)** transparency; **(L)** AC; **(M)** PC; **(N)** SLCs; and **(O)** NSLCs.

The largest effect of temperature on quality was appearance (Table 1 and Figures 4A,I–K). Either CR or CD was increased under HT (Figures 4I,J). Higher temperatures led to slight decreases in amylose and PCs (Figures 4L,M). Both starch-lipid (Figure 4N) and non-starch-lipid (Figure 4O) contents were significantly higher in all lines at higher temperature. Moreover, average taste scores of the lines were slightly higher at higher temperature (Table 1 and Figure 4B), probably due to the decreased amylose and PCs (Figures 4L,M).

### Effects of *Wx* Genotype, N-Level, and Temperature on Rice Taste and Grain Appearance

The *Wx* genotype, nitrogen level, and temperature affected the rice quality in different ways. Since grain appearance and taste score are the primary determinants of market value, we attempted to figure out the optimal combination of *Wx* genotype, nitrogen fertilization level, and temperature on taste score and grain appearance. A three-way ANOVA on rice taste score and appearance showed that the *Wx* genotype accounted for 83% of the taste score variance (Table 2); nitrogen fertilization level was the second contributory factor for taste score (*R^2^* = 0.11) and temperature had only a slight but significant effect on the taste score (*p* = 8.24E-05, *R^2^* = 0.02) (Table 2). For grain appearance (chalkiness), the *Wx* genotype was again the predominant factor (*R^2^* = 0.54) (Table 3); temperature was second (*R^2^* = 0.22), and nitrogen level had no effect on rice chalkiness (*p* = 0.47) (Table 3).

**TABLE 2:** Three-way ANOVA on rice taste score.

Taste score	Degree of freedom	Sum Sq.	Mean Sq.	*F*-value	*Pr*(>*F*)	*R* ^2^
*Wx*	12	6734.20	561.18	75.10	5.38E-30	0.82
N	2	867.27	433.64	58.03	2.24E-14	0.11
T	1	146.71	146.71	19.63	4.42E-05	0.02
Residuals	56	418.47	7.47			0.05
Total	71	8166.65				

*Wx represents Wx genotype; N, the nitrogen fertilization level; T, temperature. Sq, square deviation; Pr, p-value.*

**TABLE 3:** Three-way ANOVA on rice chalkiness rate (CR).

Chalkiness rate	Degree of freedom	Sum Sq.	Mean Sq.	*F*-value	*Pr*(>*F*)	*R* ^2^
*Wx*	10	17361.54	1736.15	10.16	9.60E-09	0.53
N	2	252.0164	126.01	0.74	0.483877	0.01
T	1	7077.703	7077.70	41.42	6.41E-08	0.22
Residuals	46	7859.483	170.86			0.24
Total	59	32550.74				

**FIGURE 5:**
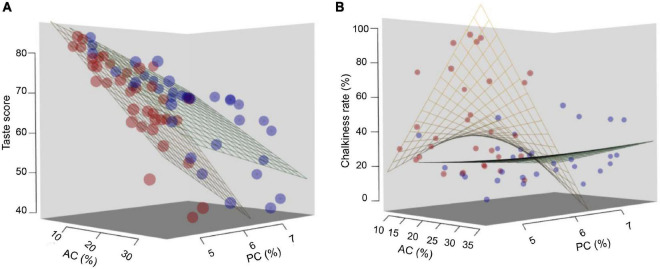
Analysis of the regression relationships on AC, PC, and temperature. Regression analyses of **(A)** taste score; **(B)** CR on AC, PC, and temperature. Red dots represent the phenotype [AC, PC, and taste score in **(A)** or CR in **(B)**] at HT and blue points represent the phenotype at normal temperature. The red and blue latticed surfaces represent the regression surfaces of the phenotypes at high and normal temperatures, respectively.

Since amylose and PCs were closely associated with the *Wx* genotype and nitrogen fertilization level, respectively, we analyzed the regression of AC, PC, and temperature with taste score and CR. As shown in Figure 5A, taste score was negatively related to amylose and PCs but not related to temperature. However, a complicated relationship between AC, PC, and temperature with CR was evident. The CRs of most lines were higher at higher temperatures. CR was positively related with AC and negatively related with PC at HT (Figure 5B). However, no such relationships were found at low temperatures (LTs) between CR and amylose or PCs (Figure 5B), implying that chalkiness was a complicated, environmentally sensitive trait.

## Discussion

### *Wx* Genotype Was the Major Determinant of Rice Quality

The *Wx* gene, which encodes granule bound starch synthase?, is the casual gene for AC in rice grain (Wang et al., 1995). Several *Wx* alleles have been identified and AC is positively related to the expression level of *Wx* (Wang et al., 1995; Bligh et al., 1998; Cai et al., 1998; Bao et al., 2002; Mikami et al., 2008). *Wx^lv^*, reported to be the ancestral allele, confers the highest AC, and functional mutations in *Wx^lv^* led to *Wx^a^*, *Wx^b^*, and *Wx^in^*, which are the most common alleles in cultivated rice (Zhang et al., 2019). *Wx^la^* was found to be a high quality *Wx* allele, conferring similar eating quality as *Wx^mp^* but with transparent appearance (Zhang C. Q. et al., 2021; Zhou et al., 2021b). Apart from the AC, *Wx* plays a key role in eating quality. Variations in GC, water absorption, and cooked rice elongation and properties were reported to be closely linked to *Wx* (Ge et al., 2005; Tian et al., 2005; Wang et al., 2007; Yan et al., 2014). Zhou et al. (2021b) reported that the function of *Wx* is associated with AC, GC, viscosity properties obtained from a rapid viscosity analyzer (RVA), and taste score. In this study, we confirmed that AC was positively related to *Wx* expression and that the *Wx* genotype was the main factor causing variation in RVA properties and taste score (Table 1 and Figure 2), a result that is consist with previous studies. We suggest that it is better to cultivate rice varieties that carry the *Wx^b^* or *Wx^la^* alleles to obtain the best quality.

### Nitrogen Fertilization and Temperature Affect Quality Differently

Nitrogen fertilization and temperature are the major factors that influence the rice quality. We found that nitrogen fertilization improved nutritional quality but at the sacrifice of eating quality (Figures 3C,J–N), and the largest effect of HT reduced appearance quality (Figures 4A, 5B and Table 3).

Nitrogen fertilizer application has been shown to increase rice yield while decreasing rice eating quality by increasing storage PC in rice grain (Gu et al., 2015; Cao et al., 2018; Liang et al., 2021). Several studies found that the starch content changed with the application of nitrogenous fertilizer. Cao et al. (2018) and Zhang et al. (2020) pointed out that AC increased with the amount of nitrogen fertilizer, but Singh et al. (2011) and Zhu et al. (2017) reported that AC decreased with an increased nitrogen level. In the present study, PC significantly increased with the nitrogen level but eating quality declined (Figures 3C,J–N) with no significant change in AC (Figure 3I). This might be due to the different timing and management of nitrogen application (Zhu et al., 2016, 2017; Zhao et al., 2020; Hu et al., 2021; Ning et al., 2021). A slight improvement in appearance quality in lines with highly expressed *Wx* alleles was observed with increased nitrogen at higher temperatures, which was consistent with a previous report (Wang et al., 2021). However, this decrease in CR and CD was not found at LT (Figures 3D–F, 5B and Table 3), suggesting the pivotal role of HTs and mutual effect among temperature, nitrogen fertilization, and *Wx* genotype on chalkiness formation.

It is generally agreed that HT induces the formation of chalky grains and reduces appearance and milling qualities (Counce et al., 2005; Zhang et al., 2016; Wang et al., 2021; Zhang H. et al., 2021). In the present study, we found that both CR and CD were higher under the higher temperature conditions and transparency decreased (Figures 4A,I–K). Other studies demonstrated that the increased expression level of α-amylases caused by HTs was the main cause of chalky grains (Hakata et al., 2012; Nakata et al., 2017). Significant decreases in amylose and PCs at HTs (Figures 4L,M) was consistent with previous studies (Sano et al., 1985; Lin et al., 2010; Zhang et al., 2016). In the present study, eating quality was better at HTs (Figure 4B), whereas earlier studies found that HTs reduced eating quality (Zhong et al., 2005; Zhang H. et al., 2021). Tang et al. (2019) suggested that nitrogen fertilization could compensate for the deterioration in rice quality caused by HT, whereas we believe that the contribution of reduced amylose and PCs to eating quality compensated for the adverse effects on eating quality. Moreover, both starch-lipid and non-starch-lipid contents were significantly higher at HTs (Figures 4N,O), implying a role of lipids in rice taste and chalkiness.

### Suggestions for Rice Quality Improvement

According to the present results, we propose that cultivars with medium amylose levels, most of which carry a *Wx^b^* or *Wx^la^* allele, cultivated with low nitrogen fertilization will always exhibit superior quality, and the avoidance of HTs will be beneficial for better appearance quality. However, global warming is occurring and nitrogen fertilization is the most effective way to maintain or increase yield despite the reduced production brought about by HTs (Peng et al., 2004; Yuan et al., 2021). Therefore, the challenge will be to breed good quality rice cultivars with tolerance to HT under optimum nitrogenous fertilization at the lowest cost of quality. Understanding the genetic basis of HT tolerance and nitrogen use efficiency will also be a promising approach to preserve yield and maintain quality.

## Conclusion

Rice quality is a complex trait determined by both genetic and environmental factors. In this study, we confirmed the predominant role of *Wx* genotype in rice quality, not only influencing eating quality, but also appearance. Nitrogen fertilization and HTs, the main environmental factors affect quality in different ways. For superior eating quality and good appearance, it is best to cultivate rice varieties carrying the *Wx^b^* or *Wx^la^* alleles. To maintain yield and high quality under HT conditions, careful consideration must be given to the level of nitrogen fertilization.

## Data Availability Statement

The original contributions presented in this study are included in the article/Supplementary Material, further inquiries can be directed to the corresponding author/s.

## Author Contributions

YH, HZ, GG, and QL proposed the concept and designed the experiment. DX, YW, and QS carried out the experiment. XY, BW, YL, PF, and ML assisted in the rice quality measurement. CZ constructed the *Wx* NIL. QZ assisted in the field management. DX and HZ analyzed the data and wrote the manuscript. All authors contributed to the article and approved the submitted version.

## Conflict of Interest

The authors declare that the research was conducted in the absence of any commercial or financial relationships that could be construed as a potential conflict of interest.

## Publisher’s Note

All claims expressed in this article are solely those of the authors and do not necessarily represent those of their affiliated organizations, or those of the publisher, the editors and the reviewers. Any product that may be evaluated in this article, or claim that may be made by its manufacturer, is not guaranteed or endorsed by the publisher.

## Supplementary Material

Supplementary Figure 1 Appearance. **(A)** Cooked rice of Nipponbare *Wx* NILs; **(B)** grain of Zhennuo *Wx* transformants at high and low temperatures; **(C)** grain of Nipponbare *Wx* NILs and **(D)** Zhennuo *Wx* transformants with different nitrogen fertilization levels at high temperature (HT).

Supplementary Table 1 Quality traits of tested materials under different conditions.

Supplementary Table 2 Daily temperature and weather conditions during flowering to harvesting period.

Supplementary Table 3 Detailed information about the *Wx* near-isogenic lines (NILs) and transgenic lines.

## Abbreviations

AC: amylose content
PC: protein content
CR: chalkiness rate
CD: chalkiness degree
HT: high temperature
LT: low temperature
SLC: starch-lipids content
NSLC: non-starch-lipids content
PKV: peak viscosity
HPV: hot paste viscosity
CPV: cold paste viscosity
SBV: setback viscosity
BDV: breakdown viscosity
CSV: consistency viscosity
GC: gel consistency
RVA: rapid viscosity analyzer.
