# Supplementary figures and images for: Effects of Wx Genotype, Nitrogen Fertilization, and Temperature on Rice Grain Quality
Source: Front Plant Sci. 2022 Jul 22;13:901541. doi: 10.3389/fpls.2022.901541 (PMC9355397; doi:10.3389/fpls.2022.901541)

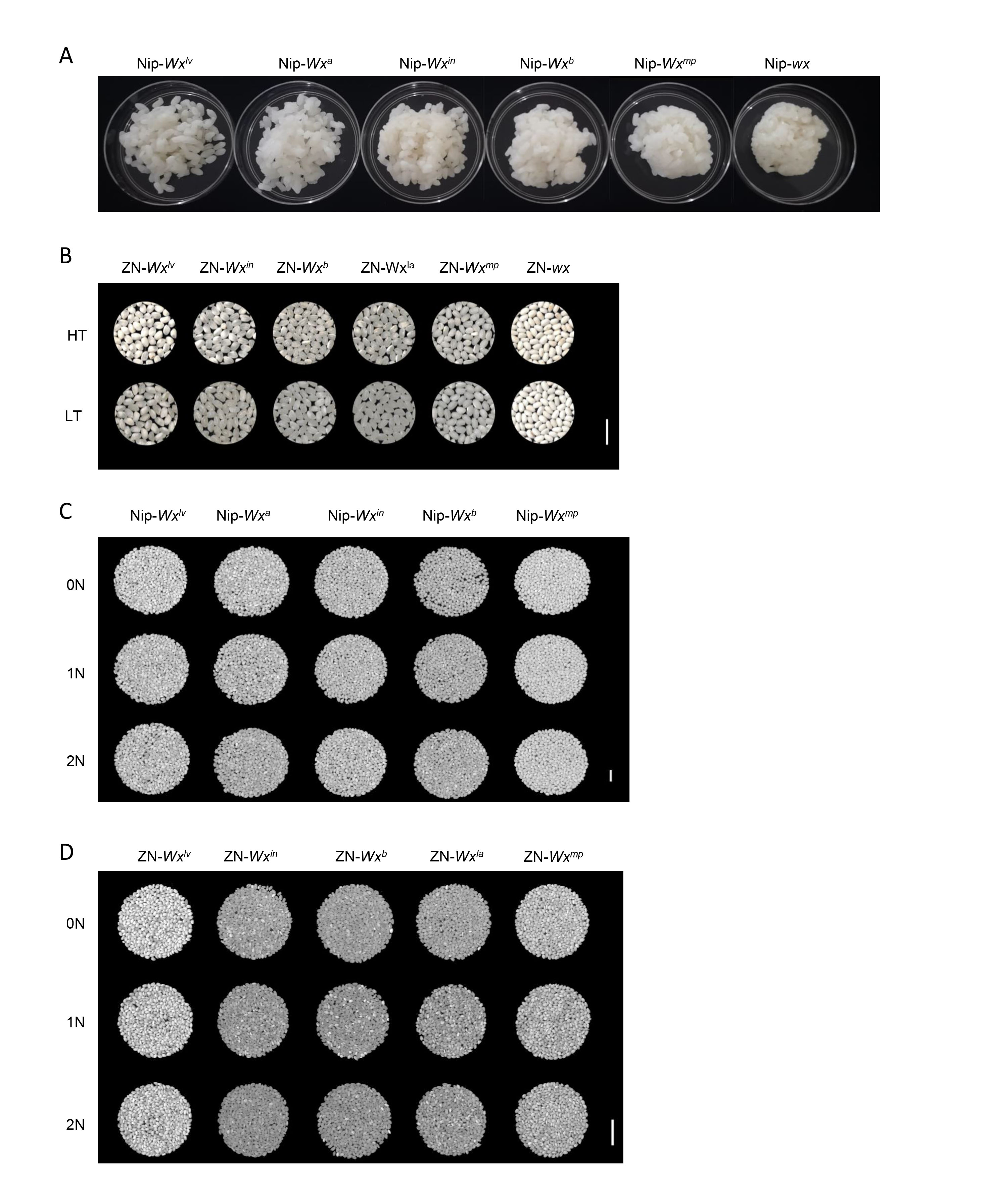

Supplement: Supplementary Figure 1 — Appearance. (A) Cooked rice of Nipponbare Wx NILs; (B) grain of Zhennuo Wx transformants at high and low temperatures; (C) grain of Nipponbare Wx NILs and (D) Zhennuo Wx transformants with different nitrogen fertilization levels at high temperature (HT). [file Image_2.JPEG]
